# Neighborhood social capital and self-rated mental health: Disparities between migrants and native residents in Beijing

**DOI:** 10.3389/fpubh.2022.1055712

**Published:** 2022-11-18

**Authors:** Xiaomeng Wang, Peiling Zhou, Zhilin Liu

**Affiliations:** ^1^School of Public Policy and Management, Tsinghua University, Beijing, China; ^2^School of Architecture, Harbin Institute of Technology (Shenzhen), Shenzhen, China

**Keywords:** mental health, neighborhood social capital, *hukou*, migrants, China

## Abstract

**Introduction:**

Although the impact of neighborhood social capital on mental health has long been recognized, the extent to which the impact differs between immigrants and local residents remains a puzzle. This study aims to bridge the gap by comparing internal migrants who are restricted by their household registration (*hukou*) status, and urban natives in China.

**Methods:**

Using self-rated mental health and social capital survey data collected in 26 neighborhoods in Beijing, this study examines the mental health outcomes of three types of neighborhood social capital, including social networks, shared norms and mutual trust, and social support.

**Results:**

The study finds that the *hukou* status of immigrants moderates the effect of neighborhood social capital on mental health, and that the internal migrants in China experience less mental health benefit of neighborhood social capital than urban natives. Compared with urban natives, neighborhood social networks have less positive effect on migrants' mental health than that of urban natives.

**Conclusion:**

The findings suggest that policy makers can improve the mental health of migrants through social capital building on the premise of eliminating the restrictions of *hukou* system on the migrants' right to participate in neighborhood activities and to access neighborhood services.

## Introduction

The mental health of immigrants has been one of the most commonly discussed public health issues across disciplines (1, 2). Studies have showed that poverty, social stigma, discrimination in the job markets, and exclusion from local public services can cause substantial mental stresses for immigrants (3–6). These risk factors for mental stress may lead to a decline in the health advantage of immigrants over time, even if they generally report superior health conditions than natives at an early stage (7, 8).

In the past few years, a considerable body of literature has investigated the benefits of neighborhood-level social capital on residents' mental health (9–11). Neighborhood social capital refers to the features of social organization within a neighborhood, such as networks, trust, and support (9, 12–14). Recent studies also found that the same formation of social capital may have different effects on individuals with different social-demographic characteristics, such as gender and income (15). However, less is known about the extent to which their mental health impacts vary between immigrants and native residents. Immigrants often are segregated in enclaves with truncated social ties and supporting system (16–20). Even when living in the same neighborhood, immigrants remain largely marginalized from the mainstream society in their everyday lives (21–23). For immigrants, living in a mixed neighborhood with urban natives does not necessarily guarantee equal access to, nor fully utilize, the “stock” of neighborhood social capital as local neighbors to maintain mental health (21, 24).

Literature on the mental health of migrant populations in Chinese cities continues to emerge (5, 6, 25–27). It was recently reported that the number of internal migrants in urban China – residents who live in cities but do not possess an official urban residency status under the household registration system (*hukou*) – had reached 236 million in 2019 (28). Like immigrants in Western cities, internal migrants in China face higher mental health risks after relocating to receiving cities (29, 30).

Unlike ethnicity-based social etiologies in most western countries, the mental health of internal migrants in China is closely related to their *hukou* status (19, 31). Migrants without a local *hukou* are restricted from enjoying universal social welfare (32–35), and experience stereotypes and stigma in their daily interactions with urban natives (23, 36, 37). *Hukou* status marks different social groups, which may be not conducive to the positive impact of neighborhood social capital on the mental health of the internal migrants. However, the disparity between migrant and native residents within Chinese cities has not been fully studied.

In this article, we extend the line of inquiry on the mental health impacts of neighborhood social capital by exploring the heterogeneous effects between migrant and native residents in Chinese cities. Based on an empirical analysis of self-rated mental health and social capital survey collected in Beijing, we test the extent to which various dimensions of neighborhood social capital (i.e., social networks, shared norms and mutual trust, and social support) predict self-rated mental health, and the extent to which these effects are moderated by individual's residency status structured by China's unique *hukou* system.

The intellectual contribution of this analysis is twofold. First, we engage with the international literature on the effects of neighborhood social capital on mental health while highlighting the differential effects across social groups, thereby providing empirical evidence to better inform community development and public health strategies. Second, we combine the emerging literature on social capital determinants of migrants' mental health with empirical studies in urban China, while highlighting the social aspects of neighborhood environment.

## Literature review

### Neighborhood social capital and immigrants' mental health

Public health scholars have sought to understand the effects of neighborhood social capital on mental health, though conceptualization and measurement of social capital have varied in different studies (9, 38). Recognizing the influences of different disciplines (39–42), public health scholars have generally viewed neighborhood social capital as multi-dimensional concept that involves interpersonal networks developed within the neighborhood, shared norms and trust, as well as reciprocal social support developed through social interactions with neighbors (9, 12).

On the one hand, neighborhood social networks, defined as informal social relationships between neighbors developed in their everyday-life encounters, can procure predictable mental health benefits (38, 43). Residents can circulate health-relevant information from social networks with neighbors, which may help enhance their own mental health (4). Further interactions with neighbors may also directly contribute to a positive psychological experience, such as a sense of belonging and security, and recognition of self-worth (44, 45).

On the other hand, shared norms and mutual trust may benefit residents' mental health by providing a source of mutual connection and mutual respect and by improving residents' sense of purpose in life (46). Neighborhood social support is also an important form of neighborhood social capital that individuals can use to cope with everyday problems (47). Neighborhood social support plays an essential role in mental health by buffering the effects of psychological stress and promoting reciprocity, especially for vulnerable groups who are more likely to rely on neighborhood resources (43, 48).

However, research in Western Europe and the United States found that immigrants may not benefit from neighborhood social capital as much as their native neighbors. First, immigrants are considered as “outsiders”, marginalized in their receiving neighborhood. They change their residences frequently, hence having difficulties in expanding neighborhood social networks or having sustained social interactions with their neighbors (15, 24). In Netherlands, Fajth and Bilgili (24) found that immigrants do not interact with their local neighbors frequently, even though they have lived in their receiving neighborhoods for many years and acquainted with most of their neighbors. Without further social interactions, the impact of neighborhood social networks on immigrants' mental health is limited.

Second, immigrants might be seen as the “others” and socially excluded by their local neighbors. Under high levels of social discrimination, frequent interactions between immigrants and native residents may lead to feelings of deprivation or mental stress (15, 49). Wutich et al. (49) research on Latino immigrants in the U.S found that those who have frequent social interactions with local neighbors report higher level of perceived stigma and worse mental health status in their ethnographic interviews. While these interactions with neighbors may also bring beneficial social capital to immigrants, i.e., trust and support, the negative emotions reinforced by neighborhood interactions may not be ameliorated by neighborhood social capital (38).

### *Hukou* system and migrants' mental health in China

Although Chinese cities do not suffer from racial or ethnic divisions seen in Western cities, internal migrants in China still experience persistent discrimination and marginalization caused in large by the *hukou* system (36, 50, 51), which may also affect the mental health of residents (5, 52). *Hukou*-based residency status may potentially moderate the effect of neighborhood social capital on mental health. First, *hukou*-based residency status creates significant disparities in local citizenship and rights, defining access to welfare and services, including healthcare services (30, 53, 54). According to Gu et al. (53) and Lao et al. (54) nationwide study (53, 54), residents with local *hukou* enjoy access to better public services, while residents without local *hukou* are denied urban citizenship and benefits that normally accompany with the citizenship. Urban natives, born with local *hukou*, enjoy full access to local public resources and services, including care for mental health (50). A small group of internal migrants, typically more educated and privileged, manage to obtain local urban *hukou* upon or after migrating to receiving cities (33). These permanent migrants with urban *hukou* enjoy similar welfare entitlement as urban natives. However, a large number of internal migrants are temporary migrants, who do not hold official urban *hukou* despite having lived and worked in receiving cities and thus deprived of equal eligibility for urban health insurance, resulting in limited access to mental healthcare resources (35).

In addition to its direct influence on mental health, the *hukou*-based residency status also indicates different accessibilities to social capital living in urban neighborhoods (26, 29, 50), which may moderate the effect of neighborhood social capital on mental health. While neighborhood social capital is demonstrated to support urban natives' mental health (26), such effects can be more complicated for migrants. Previous studies have found that temporary migrants in China are experiencing discrimination and prejudice from local urbanites and thus are excluded from local social networks (37, 55). Although migrants may maintain social networks composed of strong ties with kinship relatives and other migrants of similar places of origin (19, 21, 52), these strong yet truncated social networks may not provide diversified information and resources that are critical for a positive living experience for disadvantaged migrants, but rather reinforce a sense of marginalization and exclusion among migrants. Compared to temporary migrants, permanent migrants have developed local ties and interact with local neighbors regularly (29). However, they are still experiencing discrimination and social exclusion, despite having obtained urban *hukou* (50). Such perceived experiences of social stigma and discrimination may contribute to the psychological distress of residents (52). Additionally, while neighborhood-based social capital provides an important source of material and psychological support to get by in daily life (40, 56), bonding social capital with kinship ties can also create a strong norm of reciprocity and can sometimes become emotionally draining (56), which may further increase the psychological stress among migrants.

**Figure 1:**
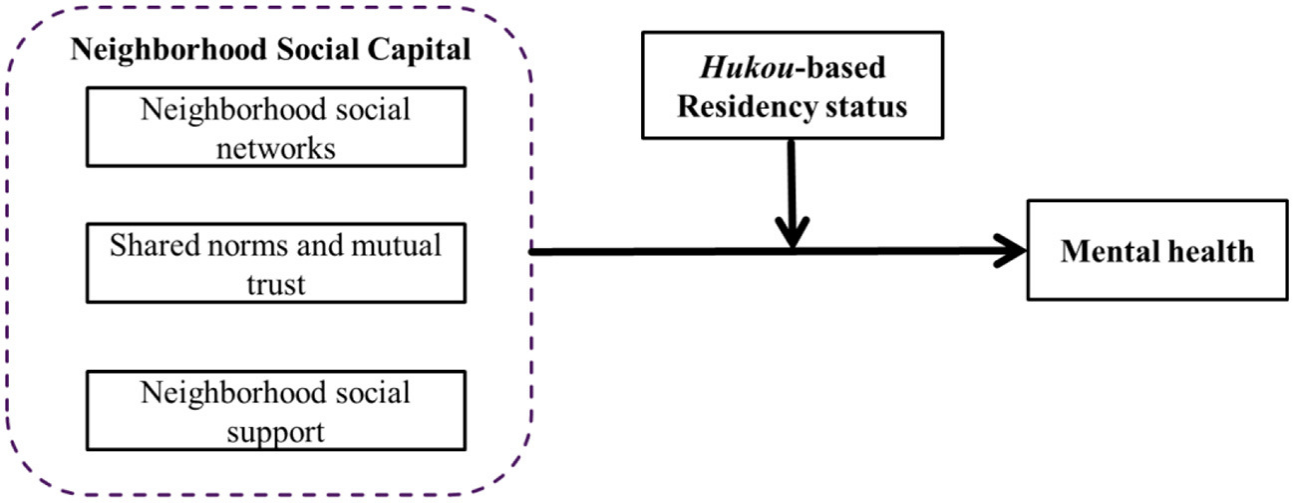
Conceptual framework.

Therefore, the empirical study aims to answer two research questions:

- The extent to which various dimensions of neighborhood social capital – neighborhood social networks, shared norms and mutual trust, and neighborhood social support – predict self-rated mental health of urban residents in China,
- To what extent the associations between neighborhood social capital and self-rated mental health are moderated by the *hukou*-based residency status (see Figure 1 for our conceptual framework).

Based on the above discussions, we hypothesize that:

- Various dimensions of neighborhood social capital – neighborhood social networks, shared norms and mutual trust, and neighborhood social support – may predict a positive mental health status for urban natives;
- The *hukou*-based residency status may moderate the effect of neighborhood social capital on self-rated mental health.

## Data and methods

### Data source

**Figure 2:**
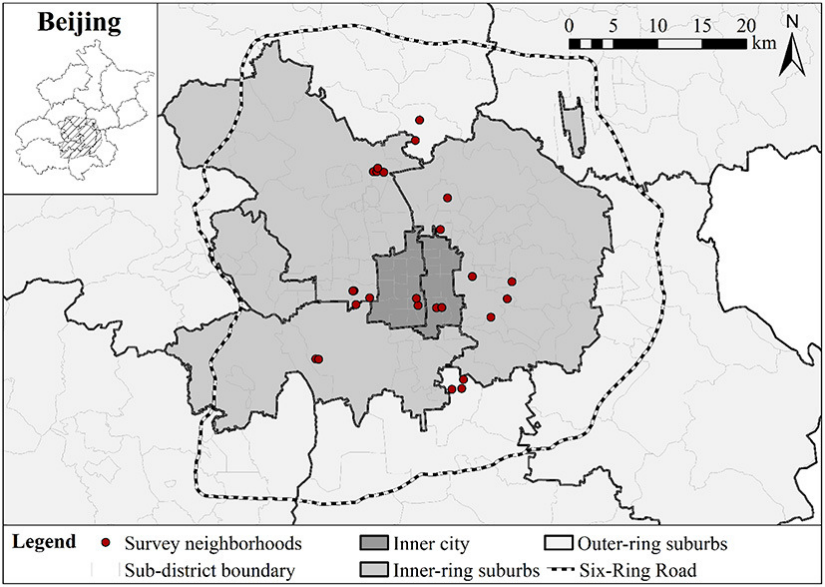
Location of the study area and survey neighborhoods.

Our data was derived from a large-scale questionnaire survey conducted in 2017 that collected the mental health and neighborhood social capital data of 1,280 residents from 26 neighborhoods (*shequ*) in Beijing, China. Beijing has seen a massive influx of migrants over the past three decades. By 2017, Beijing had about 7.94 million migrants, accounting for 36.6% of the total population (57). Chinese municipalities typically include an urbanized core area (*jianchengqu*) and rural areas in the outskirt. Our study focuses only on the urbanized area of the Beijing Municipality, which primarily is located within the Six-Ring Road (Figure 2). Following Hu et al. (58), we divided our study area into the inner-city area (*Xicheng* and *Dongcheng* districts), the inner-ring suburb (*Haidian, Chaoyang, Shijingshan*, and *Fengtai* districts), and the outer-ring suburb (parts of *Changping* and *Daxing* distrcts).

A multi-stage stratified sampling strategy was adopted to maximize the representativeness of diverse urban neighborhoods in different urban locations: four in the inner city, 16 in the inner-ring suburb, and six in the outer-ring suburb (Figure 2). From these 12 sub-districts, we selected a total of 26 neighborhoods (*shequ*) representing years of construction, housing characteristics, physical layout and environment, and socio-demographic structure. Finally, in each neighborhood we selected 50 residents (age 18–65) through a combination of systematic and stratified sampling strategies, according to the address list provided by the neighborhood resident committees. The survey eventually yielded a total of 1,280 valid samples (see Table 1 for the socio-demographic structure of the sample). To understand the effects of neighborhood social capital between migrants and urban natives on mental health, our sample did not include migrants living in other informal housing such as factory dorms or construction sites.

**Table 1:** Socio-demographic structure of the samples.

**Variable names**	**Values**	**2017 survey**		2017 official statistics^*^
		**Frequency**	**Percentage**	**Percentage**
**Residency status**	Urban natives	827	64.61%	/
	Permanent migrant	88	6.88%	/
	Temporary migrant	365	28.52%	37.2%
**Gender**	Male	626	48.91%	51.2%
	Female	654	51.09%	48.8%
**Marital status**	Married	1080	84.38%	/
	Not married	200	15.63%	/
**Education level**	Middle school or lower	216	16.88%	40.4%
	High school	375	29.30%	21.5%
	College	574	44.84%
	Postgraduate	115	8.98%	4.5%
**Employment status**	Employed	778	60.78%	/
	Unemployed	502	39.22%	/
**Co-residence**	Living with family	1111	86.80%	/
	Living alone	169	13.20%	/
**Homeownership**	Owner	957	74.77%	78.7%
	Renter	323	25.23%	21.2%
**Age**	Avg. 45.50 (S.D. 13.02)	/
**Per capita monthly household income (1000 Yuan)**	Avg. 5.52 (S.D. 13.32)	4.38

* Data from the Beijing Statistical Yearbook 2018 (57).

### Variables and measures

#### Self-rated mental health

We use self-rated score to measure the overall mental health status of the participants. The question was asked as follows in the questionnaire. ‘What do you think about your mental health in the past year in general?' The self-rated scores range from 1-very unhealthy, 2-unhealthy, 3-neutral, 4-healthy, to 5-very healthy. This measurement has been used in a number of mental health studies [e.g., (44, 59)] and has been proven valid in capturing mental health status.

#### Neighborhood social capital

We included three variables to capture different dimensions of neighborhood social capital, namely social networks, shared norms and mutual trust, and social support.

*Neighborhood social networks* was measured by the number of non-kinship ties (i.e., friends and colleagues, excluding family members or relatives) that a respondent reported to have in the neighborhood. The second variable, i.e., *shared norms and mutual trust*, was measured by a composite index derived from five 1-5 Likert scale questions asking each respondent to rate the level of agreement to five statements, including: (1) I am familiar with my neighbors, (2) people in this neighborhood have similar values and views, (3) people in the neighborhood trust each other, (4) I am able to ask neighbors for help when in trouble, and (5) people in the neighborhood get along well with each other. We calculated the Principal Component Factor score of the five items as the value of neighborhood shared norms and mutual trust (Cronbach alpha = 0.878). Finally, *neighborhood social support* was generated from a multiple-choice question in the survey that reads “if you encounter any problem in your daily life (e.g. taking care of elders or picking up children when you are not free), who is your first choice to ask for help”. The choice of “neighbors” and “residents” committee were coded as 1 and other choices (e.g. relatives and friends in other neighborhoods, or hired domestic helpers) were coded as 0.

#### The *hukou*-based residency status

Unlike most existing studies on China's internal migration, which typically treats residency status as a binary situation of migrant versus local residents, we defined residency status into three categories – urban natives, permanent migrants, and temporary migrants – to explore the nuances created by the formal *hukou* institution and the informal everyday-life experiences of migrant social exclusion. The moderating variable – residency status – was captured from two survey questions asking the birth place and current *hukou* type of each respondent. Urban natives refer to residents who are born in Beijing and with local urban *hukou*; permanent migrants refer to residents who were born outside of Beijing city but have obtained Beijing urban *hukou* after migrating to the city; and temporary migrants refer to the residents who were born outside of Beijing city and have migrated to Beijing without obtaining local urban *hukou*. Our final sample included 827 urban natives (64.7%), 88 permanent migrants (6.9%) and 365 temporary migrants (28.5%). Only a small portion of migrants is able to acquire Beijing urban *hukou* and get the identity of permanent migrants.

#### Control variables

Following previous studies about Chinese resident's mental health, Chinese resident's mental health is proved to associated with age (5, 26, 58), gender (5, 26, 58), marital status (5, 58), educational level (5, 26, 58), employment status (5, 26, 58), homeownership (5), co-residence status (5), and household income (5, 26). Therefore, we controlled eight sociodemographic variables, including age, gender (male = 1, female = 0), marital status (married = 1, others = 0), educational level, employment status (employed = 1, others = 0), homeownership (owner = 1, renter = 0), co-residence status (living with family = 1, living alone = 0), and per capita monthly household income (natural logarithm value).

### Analytic strategies

We used ordered logit regression models since our dependent variable – self-rated mental health – was rated on a 1-5 ordinal scale. Ordered logit model is utilized to estimate the probability of each categorical outcome from more than two discrete choices, in which the log odds of the outcomes are modeled as a linear combination of the predictor variables. The modeling process can be described as the estimation of coefficients β for independent variables *x* and a set of cutpoints *k*. For each sample *j*, an underlying score y^*^ was estimated as a linear function of the independent variables *x* and random error ε:


$$\begin{array}{l} y_{j}^{*}\;=\;\beta_{1}x_{1j}+\beta_{2}x_{2j}+\ldots \beta_{k}x_{kj}+\varepsilon_{j} \end{array}$$

Meanwhile, the probability of observing outcome *i* corresponds to the probability that underlying score y^*^ lies within the range of the cutpoints computed for the outcome:


$$\begin{array}{l} \text{Pr}\left(y_{j}\;=\;i\right)\;=\;Pr\left(k_{i-1}<y_{j}^{*}\leq k_{i}\right) \end{array}$$

In our ordered logit model, cluster-robust standard errors were used to account for potential heteroscedasticity due to the nested nature of the survey data, given that the samples were clustered in 26 neighborhoods. We first examine the association between self-rated mental health and neighborhood social capital and residency status with Model 1, controlling eight socio-demographic variables. In Model 2, we further include the interaction terms between residency status and three neighborhood social capital variables to test whether the associations between mental health and neighborhood social capital were moderated by residency status. To ensure the robustness of the findings, we also ran binary logit regressions by recoding self-rated mental health into a binary variable, with the values of 1–3 recoded into zero and the value of 4–5 recoded into one. The results were largely similar to those of the ordered logit regressions.

## Empirical findings

### Descriptive analysis

Table 2 shows the descriptive analysis of self-rated mental health and neighborhood social capital variables, stratified by residency status. ANOVA-tests and Chi-square tests were performed to test if significant differences exist between temporary migrants, permanent migrants, and urban natives. The self-rated mental health score of migrants (Temporary migrants: Mean = 4.160; Permanent migrants: Mean = 4.068) is slightly higher than that of urban natives (Mean = 4.017). Yet temporary migrants are significantly disadvantaged in the access to most neighborhood social capital compared with urban natives, as shown by the lower scores in all three dimensions of neighborhood social capital, although permanent migrants generally enjoy similar levels of neighborhood social capital as urban natives (Table 2).

**Table 2:** Neighborhood social capital, mental health and residency status.

**Variable**	**Full sample**	**Urban natives**	**Permanent migrants**	**Temporary migrants**	*P*-value^*^
Self-rated mental health (mean values)	4.061	4.017	4.068	4.160	0.011^**^
Neighborhood social networks (mean values)	1.406	1.477	1.388	1.246	0.411
Shared norms and mutual trust (mean values)	3.615	3.732	3.737	3.318	0.000^***^
Neighborhood social support (percentage)	34.77%	38.69%	37.50%	25.14%	0.000^***^

ANOVA are used to examine the differences between migrants and urban natives for continuous measured variables. Chi-square tests are used for categorical variables. Standard errors in parentheses:

* p < 0.1,

** p < 0.05,

*** p < 0.01.

### Association between self-rated mental health and neighborhood social capital

Table 3 presents the results of the ordered logit regression model. Model 1 presents the main effects of neighborhood social capital without the interaction terms. The average VIF value for all independent variables in model 1 was 1.61, with no VIF value of any individual independent variable exceeding 10, which indicated no serious multicollinearity issue.

**Table 3:** Ordered logit regression results: Association between neighborhood social capital and self-rated mental health.

	**Model 1**		**Model 2**	
	**OR**	**SE**	**OR**	**SE**
***Hukou*** **-based residency status (ref: urban natives)**				
Permanent migrant	0.934	0.186	0.220	0.335
Temporary migrant	0.948	0.170	0.779	0.428
**Control variables**				
Gender (reference: female)	1.213^*^	0.140	1.216	0.145
Age	0.966^***^	0.007	0.966^***^	0.007
Married	1.125	0.259	1.116	0.258
Living with family	1.294	0.283	1.296	0.280
**Education (ref: middle school or lower)**				
High school	1.250	0.262	1.272	0.266
College/university	1.238	0.285	1.263	0.284
Postgraduate	1.607	0.725	1.656	0.748
Employed	0.894	0.168	0.880	0.169
Per capital monthly household income (logged)	1.149	0.158	1.157	0.160
Homeownership (ref: renter)	0.506^***^	0.084	0.512^***^	0.086
**Neighborhood social capital**				
Neighborhood social networks	1.030	0.029	1.055^*^	0.031
Shared norms and mutual trust	0.892	0.083	0.847^*^	0.080
Neighborhood social support	1.555^**^	0.283	1.619^**^	0.333
**Interaction term**				
**Permanent migrant × Neighborhood social capital**				
Permanent migrant × Neighborhood social networks			0.916^*^	0.048
Permanent migrant × Shared norms and mutual trust			1.533	0.611
Permanent migrant × Neighborhood social support			0.905	0.365
*Temporary migrant × Neighborhood social capital*				
Temporary migrant × Neighborhood social networks			0.934^*^	0.038
Temporary migrant × Shared norms and mutual trust			1.097	0.178
Temporary migrant × Neighborhood social support			0.904	0.291
Log pseudo-likelihood	−1163.459		−1161.688	
Pseudo R^2^	0.045		0.046	
Wald chi^2^	209.355		610.108	
Prob > chi^2^	0.000		0.000	
Observations	1,108		1,108	

Robust standard errors in parentheses.

* p < 0.1,

** p < 0.05,

*** p < 0.01.

After controlling social-demographic variables, no significant difference was founded between self-rated mental health of permanent migrants, that of temporary migrants and that of urban natives. Neighborhood social support were positively associated with mental health: all else equal, a resident is more likely to report a higher mental health score if he or she perceives availability of social support in the neighborhood (OR = 1.555, *p* = 0.015; Table 3). However, neither neighborhood social networks nor mutual trust was significantly associated with self-rated mental health.

Several socio-demographic control variables were also found significant in predicting self-rated mental health (Table 3). On a 0.10 significance level, men were more likely than women to report positive mental health status (OR = 1.213, *p* = 0.093). Not surprisingly, older residents were less likely to report positive mental health status (OR = 0.966, *p* = 0.000). Interestingly, homeowners were significantly less likely than renters to report positive mental health status (OR = 0.506, *p* = 0.000), which is contrary to the findings in international literature about positive correlation between housing tenure and mental health (60). This might be related with high housing prices in Chinese large cities. Compared to renters, most of houseowners have no other options but to pay large amount of monthly mortgage after purchasing a house, therefore suffering from continuous mental stress (5).

### Moderating effects of residency status

Model 2 in Table 3 presents results from the second ordered logit regression model with the interaction terms to further test the moderating effects of residency status on the mental health outcomes of neighborhood social capital.

In model 2, as for the baseline levels of neighborhood social capital effects, all the three types of neighborhood social capital are significant associated with mental health for urban natives. In specific, urban natives with more access to neighborhood social support (OR = 1.619, *p* = 0.019) and social networks (OR = 1.055, *p* = 0.063) report higher level of positive mental health status. In contrast, those urban natives perceiving higher level of mutual trust and shared norms report worse mental health status (OR = 0.847, *p* = 0.079). The significance of neighborhood social support is consistent with model 1, which is more robust than social networks and shared norms and mutual trust.

**Figure 3:**
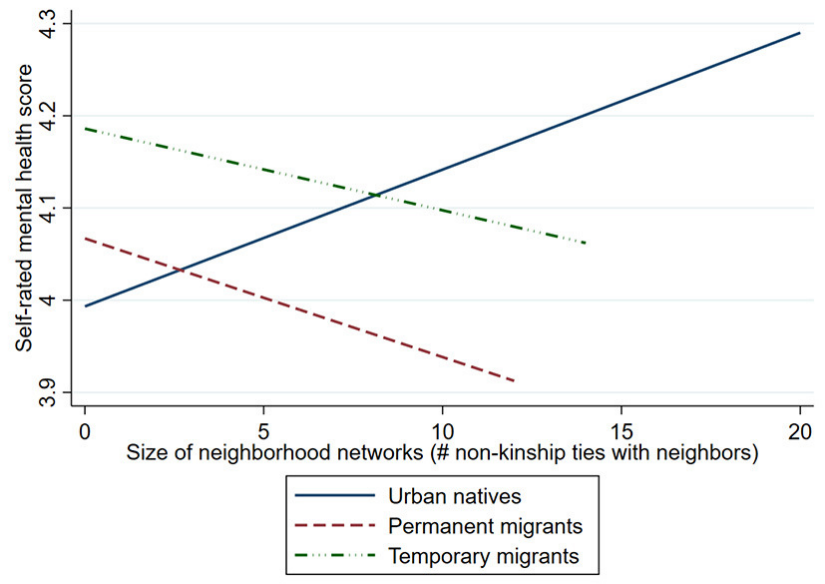
Predicted relationships between neighborhood social capital and self-rated mental health by different residency status.

There is significant difference in the mental health impact of neighborhood social networks between migrants and native residents. Model results also indicate that resident status significantly moderates the mental health impacts for one dimension of neighborhood social capital – i.e., social networks, but not for neighborhood social support and shared norms and trust. Migrants are less likely to report the same level of self-rated mental health as urban natives, despite having the same size of neighborhood social networks. As shown in in Table 3, the OR for the interaction term between neighborhood social networks and residency status is 0.916 (*p* = 0.095) for permanent migrants and is 0.934 (*p* = 0.089) for temporary migrants. In other words, whereas urban natives are more likely to report better mental health with increased neighborhood social networks, such a positive association is significantly reduced for permanent and temporary migrants. Figure 3 illustrates the estimated associations between neighborhood social networks and respondents' self-rated mental health across different residency status. For both permanent and temporary migrants, larger neighborhood social networks predict lower level of self-rated mental health. We also estimated the difference between two migrant subgroups by setting permanent migrants as the baseline reference in interaction terms. The OR of interaction term reveals a stronger effect of neighborhood social networks for temporary migrants compared to permanent migrants, but the difference is not significant.

## Discussion and conclusion

Based on the cross-sectional questionnaire survey data of 26 neighborhoods in Beijing, China, this study examines the heterogenous effects of neighborhood social capital on the mental health between migrants and urban natives in the institutional context of the *hukou* system. Although migrants may have access to formal housing in socially mixed urban neighborhoods, they have relatively less neighborhood social capital than their native neighbors. The residency status of migrants plays a negative role in moderating the association between neighborhood social capital and mental health.

The positive effect of neighborhood social support and social networks on mental health for urban natives are consistent with previous findings (11, 26, 27). Comparatively, shared norms and mutual trust is negatively associated with mental health for urban natives, which is consistent with some findings in previous studies (15). This potential downside of social capital is more obvious in the context of population fluidity. For instance, one study in Japan found that the gap between positive mutual trust and negative experience of social interaction with dissimilar neighbors may cause psychological distress, which in turn affect mental health (61). Chinese urban neighborhoods have experienced the disbanding of social relationships over the years, partly due to the influx of migrants (62, 63). Compared with internal migrants, urban natives are more likely to have positive expectation on mutual trust and thus experiencing a significant gap between positive mutual trust and negative experience of social interaction with dissimilar neighbors (64, 65), which leads to poor self-rated mental health.

According to the second model which examines the interaction terms between residency status and three neighborhood social capital variables, immigrants did not gain as many mental health benefits from increased neighborhood social networks as their native neighbors. While previous studies found the importance of neighborhood social networks in improving mental health outcomes (11, 38, 43), this study suggests that the mental health benefits of neighborhood social networks can be attenuated by particular *hukou*-based residency status. Temporary migrants without local *hukou* may create stereotypes in the minds of urban natives, leading to self-isolation behaviors and avoidance of interaction with natives (36). Although some migrants have already developed social networks in their neighborhoods, their interaction with neighbors are relatively limited in improving their mental wellbeings (18). For those migrants who feel social marginalized and institutionally excluded, building greater social networks with local neighbors does not confer mental health benefits, but rather negative perceptions of social status (21) leading to worsening mental health outcomes. Despite having local *hukou*, permanent migrants who have not lived long enough in local urban society are less likely to develop close ties and interactions with the receiving neighborhoods (66). Their interactions with neighbors tend to be instrumental rather than emotional, which does not do much help to relieve their stress or anxiety, and thus appears to be less effective in improving mental health (67).

This research has several policy implications. Firstly, in parallel with other studies in Western countries, our findings indicate that neighborhood social capitals can make a significant contribution to residents' mental health. Therefore, to make residential areas a healing place for promoting mental wellness, planners and policy makers should pay more attention to the neighborhood social environment and foster a friendly neighborhood atmosphere. Secondly, migrants, especially temporary migrants without local *hukou*, are vulnerable to the negative impacts of neighborhood social capital on mental health. Therefore, this study calls on social organizations in local neighborhoods to abandon the restrictions of the household registration system, and provide permanent and temporary migrants with more opportunities to involve in neighborhood collectives and foster a sense of belonging. Building a healthy city for the mental wellness of all citizens requires integration between urban natives and immigrants, which needs the supports from China's macro-level institutions and micro-level community development efforts.

Several limitations of this study need to be acknowledged. First, our study relied on cross-sectional survey data. It is difficult to either infer causality from association, or to fully capture the multi-faceted causal mechanisms of neighborhood social capital and mental health. Longitudinal data and ethnographic materials with more detailed information should be used in future research. Second, with Beijing as research site, other types of Chinese cities, including medium-sized cities, were not considered. As the household registration policy is less stringent, the situation of immigrants in medium-sized cites might be different. Therefore, it is necessary to conduct a cross-city study to better understand the effects of neighborhood social capital on mental health of migrants in urban China. Finally, using one self-rated question to measure mental health has limitations in accuracy compared to using standard mental health questionnaires such as PHQ-9. The use of standard mental health questionnaires is thus encouraged to precisely assess mental health status.

In conclusion, this study finds neighborhood social capital has different effects on the mental health of migrants than urban natives in mixed urban neighborhoods. Our empirical evidence engages in the theoretical debate about how the effect of neighborhood social capitals on immigrant mental health are attenuated, extending the implications of neighborhood social capital in the migrant and mental health literature. In future research, a mixed-method approach should be taken to better understand the specific mechanisms by which individual life experiences, neighborhood social capital, and macro-social institutions jointly determine the mental health of floating populations.

## Data availability statement

The datasets presented in this article are not readily available because the data collected for this study is confidential. Requests to access the datasets should be directed to ZL, zhilinliu@tsinghua.edu.cn.

## Ethics statement

This study does not involve medical experiments. Ethical approval for the survey was obtained from the Ethics Committee of the School of Public Policy and Management, Tsinghua University. Informed, oral consent was obtained from all survey participants. Written consent for participation was not required for this study in accordance with national legislation and the institutional requirement.

## Author contributions

XW, PZ, and ZL: study conception, design, and analysis and interpretation of data. XW and ZL: acquisition of data. XW and PZ: drafting of manuscript. PZ and ZL: critical revision. All authors contributed to the article and approved the submitted version.

## Funding

This research was supported by the National Natural Science Foundation of China under Grant Number 41901185 and 4207120. This research also received support from the Shenzhen Science and Technology Innovation Commission under Grant number GXWD20201230155427003-20200821180448001, and the Startup Foundation for High-Level Talents of Harbin Institute of Technology (Shenzhen).

## Conflict of interest

The authors declare that the research was conducted in the absence of any commercial or financial relationships that could be construed as a potential conflict of interest.

## Publisher's note

All claims expressed in this article are solely those of the authors and do not necessarily represent those of their affiliated organizations, or those of the publisher, the editors and the reviewers. Any product that may be evaluated in this article, or claim that may be made by its manufacturer, is not guaranteed or endorsed by the publisher.
